# Cancer-Induced Reprogramming of Host Glucose Metabolism: “Vicious Cycle” Supporting Cancer Progression

**DOI:** 10.3389/fonc.2019.00218

**Published:** 2019-04-04

**Authors:** Polina Schwartsburd

**Affiliations:** Institute of Theoretical and Experimental Biophysics, Russian Academy of Sciences, Pushchino, Russia

**Keywords:** cancer biology, glucose, insulin resistance, metabolism, pregnancy, stress

## Abstract

Unrestricted cancer growth requires permanent supply of glucose that can be obtained from cancer-mediated reprogramming of glucose metabolism in the cancer-bearing host. The pathological mechanisms by which cancer cells exert their negative influence on host glucose metabolism are largely unknown. This paper proposes a mechanism of metabolic and hormonal changes that may favor glucose delivery to tumor (not host) cells by creating a cancer-host “vicious cycle” whose prolonged action drives cancer progression and promotes host cachexia. To verify this hypothesis, a feedback model of host-cancer interactions that create the “vicious cycle” via cancer-induced reprogramming of host glucose metabolism is proposed. This model is capable of answering some crucial questions as to how anabolic cancer cells can reprogram the systemic glucose metabolism and why these pathways were not observed in pregnancy. The current paper helps to better understanding a pathogenesis of cancer progression and identify hormonal/metabolic targets for anti-cancer treatment.

## Introduction

Metabolism consists of catabolic processes, i.e., the breakdown of molecules resulting in the release of energy, and anabolic processes, i.e., the synthesis of predecessors and components for proteins, lipids, and nucleic acids which consumes energy. Maintenance of the delicate balance between anabolism and catabolism is one of the most important requirements for organism survival, especially in critical host situations, such as embryo growth in pregnancy or unrestrained cancer proliferation. Glucose is an essential fuel for embryo and tumor cells wherein glucose uptake is independent of insulin. This contrasts with glucose uptake by insulin-dependent cells, such as skeletal muscle, fat and hepatic cells. Reduction in the ability of these cells to take glucose from the blood in response to normal circulating levels of insulin is known as insulin resistance (IR).Chronic IR is a key pathological feature of obesity, type 2 diabetes with an increased weight of the patients and cancer cachexia with a reduced weight of the cancer-bearing host (1). A possible explanation for this paradox is that the IR is a two-sided mechanism acting under opposite catabolic and anabolic conditions (2, 3). This hypothesis then raised the important question about tumor-host redistribution of glucose supply between anabolic cancer cells and catabolic host cells with chronic IR. Another fundamental question that remains to be answered concerns the key differences between glucose-controlled pathways in pregnancy and in the tumor-bearing host. What controls the metabolic and hormonal differences between fetal- and cancer-induced IR? A comparison of the adaptive and pathology responses of IR that can support the anabolic growth of embryo or cancer cells is presented in this opinion article. What metabolic changes in glucose homeostasis may lead to transition from adaptive to chronic IR in the analyzed cases? What are possible pathological consequences of such a transition in cancer-supported IR, and can they lead to cancer progression? What determines this chronic insulin resistant state and how can it be overcome? The purpose of this work is search for answers to these questions.

## Insulin Resistance During Pregnancy and Cancer: from Adaptive Response to Pathological Cancer Cachexia

The survival of multi-cellular organisms depends on the organism ability to maintain glucose homeostasis for the time of low/high nutrient availability or high glucose requirement to support cellular proliferation. Glucose is an essential fuel for cellular growth in adaptive responses (such as pregnancy and immune protective response) or in uncontrolled proliferative cancer disease. Pregnancy is a period marked with adaptive changes in the women's hormonal status and metabolism. The ability to regulate nutrient balance during this period is critical for health of the mother and the growing fetus. It is well known that glucose is the primary source of energy and structural materials for the embryo growth during pregnancy. Embryonic consumption of glucose passes ahead of glucose availability from the pregnant mother. Because of this, the normal concentration of blood glucose in the embryo/fetus is lower by 10–20 mg/100 ml (0.6–1.1 mmol/l) than in the blood from the pregnant mother (3.3–6.6 mmol/l). This difference becomes maximal in the second-half period of human pregnancy. It is interesting to note that this period is characterized by physiological IR (4) that is induced only in mother cells primarily as adipocytes and skeletal muscles (5). The reproductive hormones and cortisol do not significantly correlate with the change in insulin sensitivity during pregnancy, in contrast to TNF-α (6). Such host compensatory IR can be regarded as an additional possibility for maintenance of the sufficient glucose consumption by the fetus through re-distribution of the unused glucose supply from maternal tissues with IR to fast-growing fetal tissues (7). Moreover, this effect is temporary and can be controlled by the placenta, in which TNF-α and leptin are produced, and therefore could play a central role in IR during human pregnancy (8, 9). Most of the placental TNFα (94%) is released into the maternal circulation and only 6% is released to the fetal side (6). Therefore, it seems plausible that an elevated level of maternal TNF-α could attenuate insulin signaling through a decrease in insulin-stimulated phosphorylation of the insulin receptors and its substrate (10), thus causing the decreased insulin sensitivity and glucose uptake within woman muscle and fat tissues. As a result, the reserved glucose resources become more available for embryonic cells that require an additional glucose supply for their fast growth. This compensatory effect can result from the ability of the fetal cells to uptake glucose without insulin support (11).

**Figure 1:**
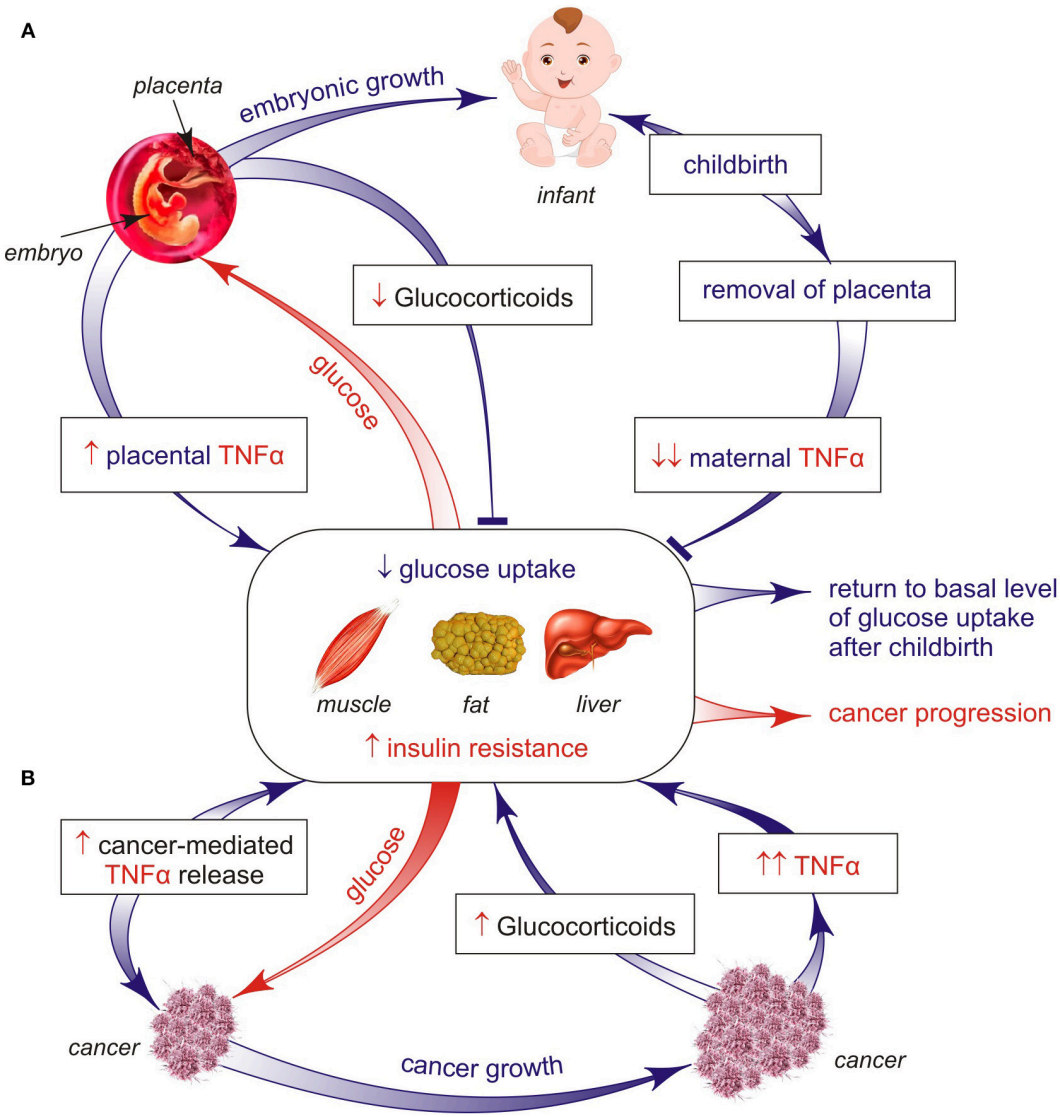
**(A,B)** Two different processes (late pregnancy and cancer) invoke a similar insulin-resistant response, but with apparent opposite impacts. Both types of IR are a secondary but necessary development to counteract hypoglycemia in pregnant women and tumor-bearing hosts. One key mechanism that may explain the origin of pregnancy-controlled IR is based on the placenta ability to release TNFα cytokine, the most part of which is infused into the mother's (not fetus's) blood, causing the TNFα-induced inhibition of glucose uptake by woman's organs, such as muscles, fat, and liver. As a result, this unused glucose can come to the fetus that badly needs glucose as the basic fuel for its growth. This mechanism is reversible because placenta-controlled induction of the maternal IR is restored to the basal state after childbirth and placenta removal. Such adaptive mechanism can be irreversibly converted to persisted IR when uncontrolled inflammation and chronic stress develop in cancer-bearing hosts, both being related to catabolic programs causing cachexia via the loss of muscles and/or fat mass and pancreatic beta-cell failure. As a result, the efficiency of two distinct forms of the insulin resistant mechanism both controlling host glucose concentrations has contrasting impacts on host homeostasis, namely, one mechanism supports the generation of a new fetus life, whereas the other mechanism increases the risk of developing cancer cachexia leading to higher mortality.

After childbirth, the concentration of the maternal TNF-α reduces rapidly to the normal basal state, in parallel with declined IR to its original state which is typical of healthy women (Figure 1A). However, the inadequate high intake of dietary nutrients is associated with the development of IR in the offspring later in life (12). In fact, an obese pregnant woman has subclinical endotoxemia associated with increased IR and pro-inflammatory cytokines to a greater extent than a non-obese pregnant woman (12). As a consequence, after childbirth the overweight pregnant woman often does not restore the IR, which results in an increased risk to the advent of diabetes in future. However, a rigid quality control assures reversion of placenta-controlled IR and can keep an adequate fetus growth during physiological pregnancy. As cancer is a highly glucose-demanding tissue, could a similar cell growth-supporting mechanism also operate in cancer-bearing animals?

Different aggressive low-differentiated cancer cells show a fetal characteristics, among which are induction of some fetal isoforms of proteins and antigens (13, 14). These findings form the basis for the concept that “oncogenesis is a partially blocked ontogenesis” (15). One common feature of cancer and embryonic cells is that they use glucose as the key source of cellular fuel. It is common knowledge that the glucose uptake rate becomes dramatically enhanced when cells acquire malignant properties. The earliest known metabolic abnormality associated with cancer was glucose intolerance (16). In this condition most of cancer cells consume glucose with a higher rate than insulin-controlled organs in the cancer-bearing host. It is significant that chronic IR is noted in malignant, but not benign tumors (17). Using the gold standard hyperinsulinemic-euglycemic clamp technique for measuring insulin sensitivity, peripheral IR was recognized in patients with colorectal (18), lung (19) and other types of malignancies (20, 21). It should be noted that cancer-induced IR was not associated with the disease stage or the degree of weight loss, but was weakly associated with the degree of host inflammation. An experimental study on mice with colon tumors (22) also confirmed the conclusion that IR was observed prior to the development of weight loss. In other words, periphery IR is a common characteristic of various tumor-bearing hosts, but after surgical removal of the tumor the level of insulin sensitivity has been restored (23). Such IR is characterized by diminished responsiveness of muscle and fat tissues to the insulin-controlled glucose uptake that is usually associated with a compensatory rise of insulin pancreatic production and its release into the blood in tumor-bearing hosts (23). Chronic hyperinsulinemia and IR can create a supporting condition to accelerate tumor growth, probably via increased activation of the phosphatidylinositol 3-kinase pathway (24, 25). Moreover, the sensory threshold of the insulin-controlled glucose uptake in the muscle/fat cells is significantly elevated during multi-step cancer progression, for example, in transition from hyperplasic lesions to islet tumors (26), but together with an increased risk of circulating glucose deficit. One cause of this dangerous state is aerobic glycolysis (Warburg effect) that is the main characteristic feature of glycolytic cancer cells, i.e., a continuous high glucose uptake and a higher rate of glycolysis leading to increased lactate production in glycolytic cancer cells as compared to normal cells. Moreover lactate can be imported into oxidative cancer cells that use lactate in mitochondrial metabolism as a main fuel compared to glucose, thus sparing glucose for glycolytic cancer cells (27, 28). The Warburg effect helps glycolytic cancer cells to produce energy and biosynthetic precursors for their uncontrolled proliferation but it might increase a risk of developing hypoglycemia by fast growth of some aggressive cancers (personal observation). A way to decrease this pathological state is the compensatory activation of hepatic gluconeogenesis—endogenous glucose synthesis *in vivo*. Different gluconeogenic precursors, such as lactate, alanine, and glycerol, contributing to prevention of systemic glucose deficit can be used. As an example, cancer-produced lactate is recycled to glucose by the liver through the ineffective Cori cycle. This extensive glucose recycling accompanied by increased energy expenditure in tumor-bearing hosts has been documented particularly in cancer patients (29). It is well known that induction of IR in the liver is also associated with activation of gluconeogenesis, as in fasting, diabetes, or cancer growth (30, 31). What pathways and factors could be responsible for the induction of IR in cancer-bearing hosts and what consequences can be expected?

**Figure 2:**
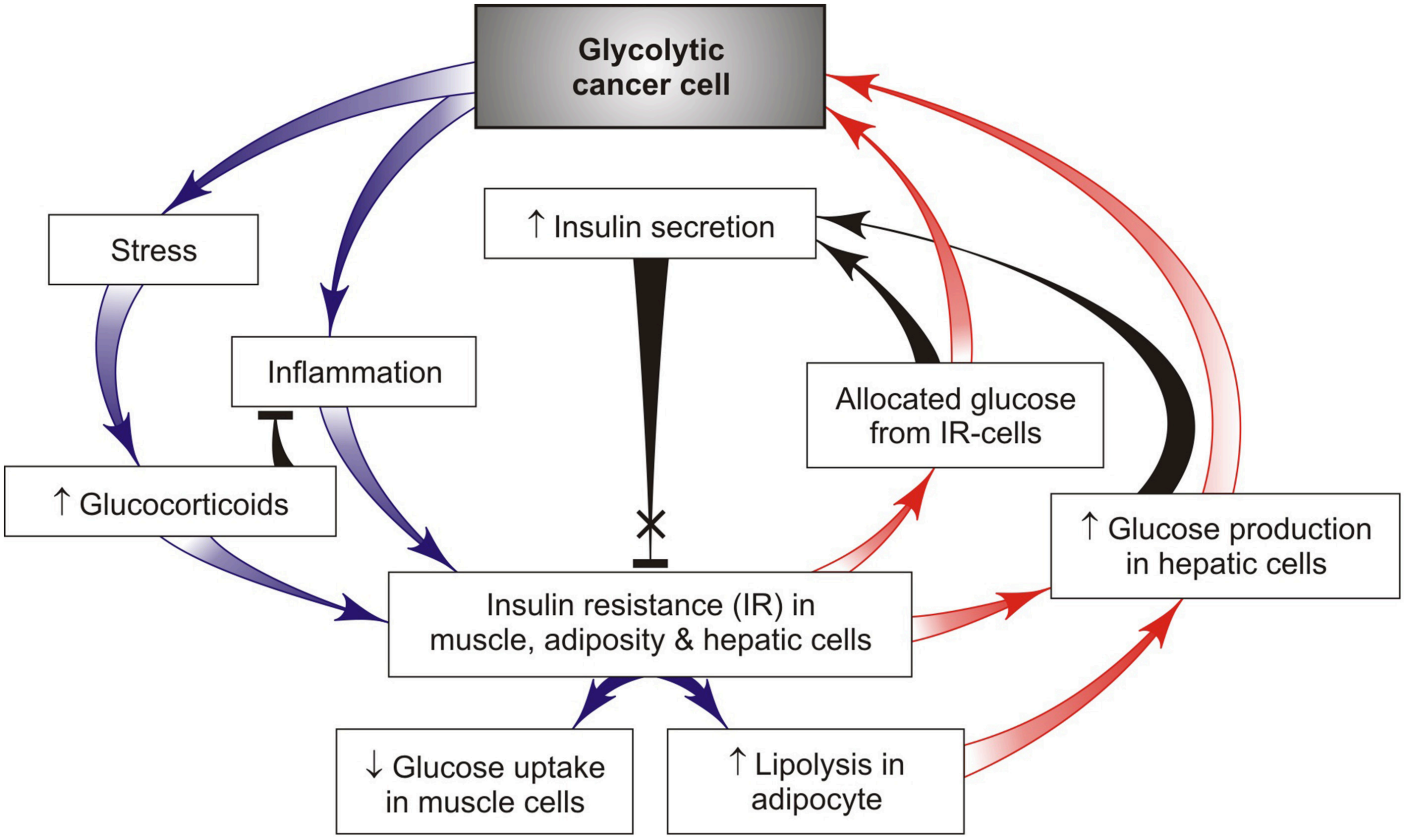
A simple feedback model of tumor-host metabolic interactions capable of creating a “vicious cycle” that promotes cancer growth through chronic activation of hepatic gluconeogenesis and redirects the available glucose from insulin-resistant tissues such as skeletal muscle, fat, and liver, to glycolytic cancer cells (red arrows). This effect is achieved by cancer-induced activation of chronic stress and systemic low-grade inflammation that support chronic IR in host tissues and act as the driver of such metabolic alterations promoting cancer progression.

Previous studies have reported that the main catabolic pathways including lipolysis, inflammation and stress (Figure 2) can play a crucial role in development of IR in obesity, type 2 diabetes and cancer (17, 32). Factors released by tumor-induced lipolysis in the white adipose tissue are accompanied by elevated levels of systemic free fatty acids (FFAs) that are able to restrict glucose utilization and induce IR in skeletal muscles (33). These findings suggest that this reserved glucose comes from muscles with IR into cancer cells for the maintenance of their survival and growth. The opposite effect can be expected from host insulin-sensitive cells due to the glucose and insulin deficit. There are some sensitive target examples of such tissue-specific alterations. One of them is fast-twitch glycolytic myobibers in white muscles that are susceptible to fatigue by induction of muscle IR, in contrast to slow-twitch oxidative myofibers in red muscles that are resistant to fatigue (34). Therefore, it is not surprising that the chronic maintenance of IR in a muscle results in the loss of its function together with the elevated muscle fatigue in cancer-bearing hosts. Another example is the effector T-lymphocytes and dendritic cells that require increased glucose levels for glycolysis to produce anti-cancer cytokines (35). As a result, under conditions of glucose deficit immune cells *in vivo* lose their anti-cancer activity, in contrast to naive and immunosuppressive T-cells that mainly utilize fatty acids to support their activity and tumor growth (36). It is of interest that the lymphoma-mediated defect in interferon production could be rescued simply by the addition of glucose to CD4 T cells *in vitro* (35).

Stress and inflammatory pathways are critical in the mechanisms underlying IR (17, 37) and beta-cell dysfunction lowering insulin production (38). Inflammation is generally associated with the catabolic state, leading to glucose mobilization to combat infection and other stressors such as cancer (but only if inflammatory response acts locally and intensively within the tumor). Inflammatory cytokines are signals from cancer tissues to induce catabolic responses in insulin-controlled adipose and muscle cells that potentiate their lipolysis, proteolysis, and IR (37). Stress and inflammatory activated lipolysis and proteolysis lead to the appearance of gluconeogenesis precursors, such as glycerol and amine acids, that support the endogenous glucose synthesis in liver due to cancer development (1). Under these conditions, tumor-bearing hosts use the reserves from adipose and skeletal tissues for hepatic glucose synthesis that maintains cancer growth. Such conditions are still retained in cancer progression associated with an increase in pro-inflammatory cytokines TNFα and IL-6 maintaining systemic IR (Figure 2). It is significant that IR occurs *before* the beginning of muscle and fat weight loss (22). A bidirectional signal exchange between such systemic inflammation and IR might induce a self-sustained vicious cycle (Figures 1B, 2) where chronic IR/inflammation helps to redirect the increased glucose influx toward cancer (not host) cells and in this way supports cancer progression. This glucose rise is accompanied by compensatory higher insulin releases from pancreatic beta-cells for the purpose of inhibition of lipolysis and gluconeogenesis. However, the potential of prolonged insulin release at later stages of the disease is limited, which may lead to hypoinsulinemia (39–41). This conclusion has been supported by leukemic tumor studies (41). In these conditions, glucagon, a well-known catabolic hormone and insulin antagonist, is amply released (42, 43). All these changes can act as a pathological basis for cancer progression and increase the risk of developing the cancer cachexia.

Cancer cachexia and pregnancy are two different conditions in terms of energy balance. Cachexia is the status of a negative energy balance due to the increased energy expenditure that is accompanied by catabolic-supported IR with insulin deficit, reduced food intake, and muscle, pancreatic gland, liver and fat dysfunctions. In contrast, pregnancy is characterized by the positive energy balance and transient maternal IR with sufficient insulin secretion (44). However, the common feature in the two states is inflammation and IR that is controlled via the placenta only at late pregnancy, in contrast to cancer where it is permanent. In cachexia, the uncontrolled elevation of IL-6 and TNF-α is enough to trigger an obvious increase in energy expenditure leading to muscle and fat loss. During pregnancy, although inflammatory cytokines are released by the placenta, the increase is not sufficient to induce muscle and fat loss. In the two states, although inflammation is of different degrees, the role of adaptive/chronic IR and pro-inflammatory cytokines remains identical in the induction of energy expenditure required for embryo or cancer growth.

## Cancer-Induced Distance Alterations in Host Glucose Metabolism As Targets For Anti-Cancer Treatment

It is well known that the majority of cancer cells exhibit increased rates of glucose uptake and glycolysis as compared to non-proliferating normal tissues. This effect was first described more than 90 years ago by Otto Warburg. For chronic maintenance of increased consumption of glucose by cancer, the transformed cells generate a parasite-like behavior that deprives normal cells in multiple organs of glycolytic fuel while increasing glucose availability to cancer cells. This pathology effect can be achieved by different pathways, including chronic induction of insulin resistant state in muscle, liver and adipose tissue. An example is leukemic cells that induce production by the adipose tissue of the insulin-like growth factor-binding protein 1 (IGFBP-1), which subsequently supports tumor cell proliferation and promotes host IR while reducing circulating insulin, increasing glucose availability to the cancer cells (17, 41). Treatment with blocking antibody or agonist receptor for IGFBP-1 is able of redirecting the systemic glucose flow from leukemic cells to host tissues, thus increasing survival of leukemic mice (17).

However, cancer cells have developed another strategy that could give them, but not the host organism, an advantage in the glucose supply. This result could be achieved by a distant cancer-induced stress- and inflammatory response that gives rise to peripheral IR responsible for activation of hepatic glucose synthesis and redirection of unused glucose from muscle and adipocytes with IR to glycolytic cancer cells. The pathways integrating stress and inflammatory response with insulin action are detailed in Figure 2 that helps to indicate the key targets required for the maintenance of increasing glucose resources available for cancer cell growth through formation of the cancer-host vicious cycle.

**Figure 3:**
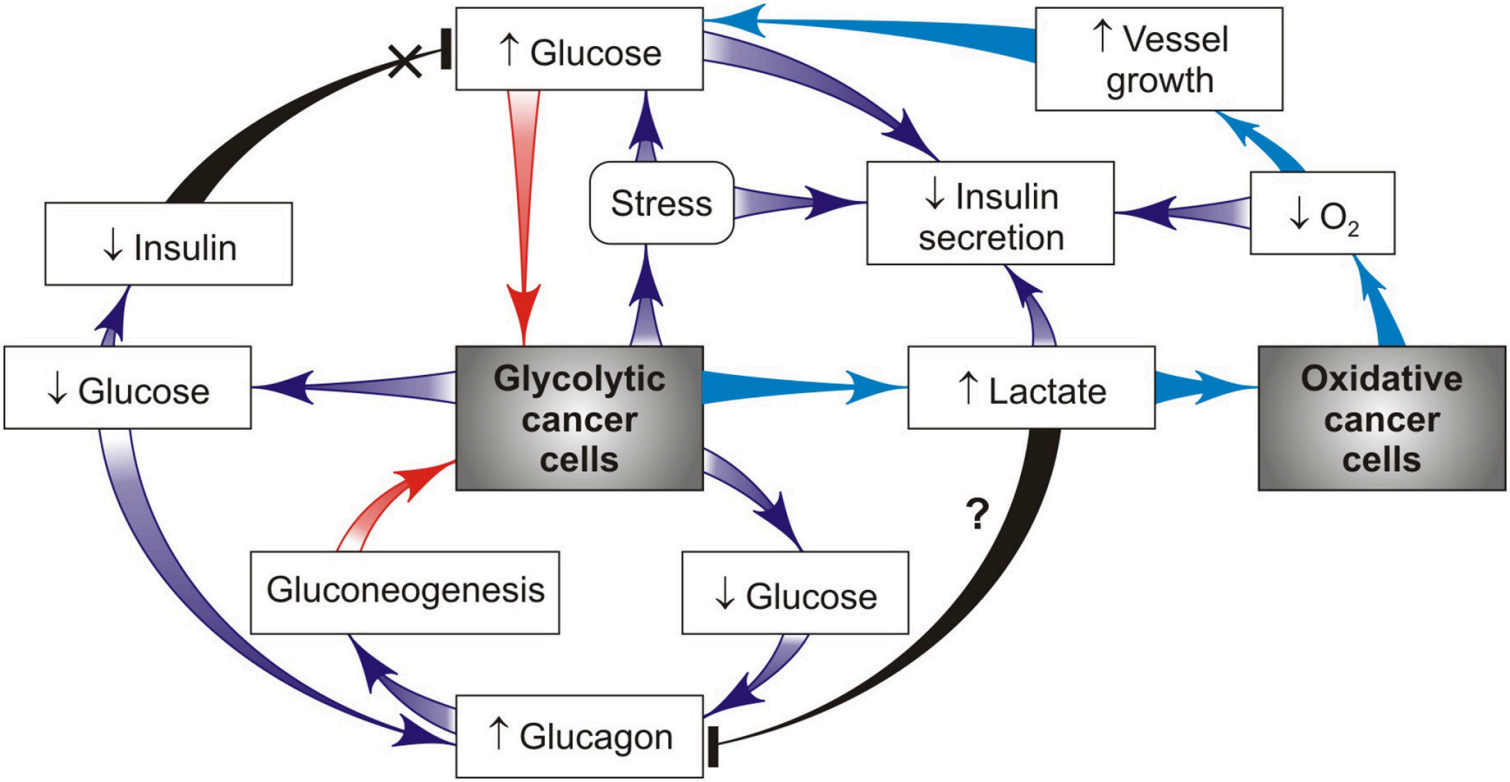
Cancer-host interactions create a hormone alterations-supported vicious cycle that drives priority glucose supply to glycolytic cancer cells (red arrows). This effect is realized through glucagon-stimulated gluconeogenesis and/or glucocorticoid mobilization of blood glucose, import glucose and then sequentially convert glucose to ATP and lactate using glycolysis. According to the ≪metabolic symbiosis≫ hypothesis (26), lactate produced by glycolytic cancer cells is imported by oxidative cancer cells that use lactate in mitochondrial metabolism as a main fuel compared to glucose, thus sparing glucose for glycolytic cancer cells (such pathway is shown in blue).

Glucose homeostasis is also regulated systemically by insulin and glucagon acting in the opposite direction (Figure 3). In healthy individuals, the level of plasma insulin level increases during hyper-glycemia, which is aimed to reduce the hepatic glucose output and to promote glucose utilization and/or disposal in peripheral tissues. In contrast, glucagon is secreted during hypoglycemia to increase the hepatic glucose output, thereby restoring the normal glucose level. Together with glucocorticoids, the insulin-to-glucagon ratio acts as a hormonal rheostat controlling glucose homeostasis in healthy individuals. This hormonal ratio in cancer patients varies over a large range depending on cancer stages. A rise in circulating glucose and insulin levels often occurs as an early stage as in breast cancer development along with appearance of IR (26). In this period glycolytic cancer cells need a large glucose amount that can come from insulin-resistant tissues (Figure 1) and hepatic gluconeogenesis (Figure 2). The opposite trend was observed in the chronic phase of the disease that is attended with prolonged stress and inflammation leading to decreased insulin secretion (41) and increased production of catabolic hormones such as glucagon and/or stress glucocorticoid (Figure 3). Maintaining the balance between these hormones is necessary for retention of blood glucose in the normal range or its minor reduction (40, 43). Figure 3 presents a simplified model showing how cancer cells can alter insulin, glucagon and glucocorticoid secretion to ensure their priority in glucose supply. Correction of the levels of these hormones can be considered as a target for preventing cancer progression. Indeed, in patients with gastrointestinal cancer, daily low-dose insulin treatment resulted in significant improvement of micronutrient intake and fat metabolism (e.g., decreased serum FFAs and increased whole body fat, particularly in the trunk and leg compartments), without indications that insulin stimulates cancer progression (20). Additional evidence from the animal models supports these findings. It was demonstrated that animals with implanted Walker 256 tumor showed improvement in the cachectic symptom after daily treatment with low doses of insulin. This positive effect could be attributed to the prevention of fat and body mass loss, in part by insulin-induced inhibition of lipolysis and anorexia (40). The insulin treatment also significantly decreased the leukemic mice burden (41). When combined with anti-inflammatory indomethacin, insulin alleviated cancer cachexia symptoms in the mouse model with implanted colon-26 adenocarcinoma better than insulin alone (45). However, the glucose-lowering ability of the insulin therapy has a side effect (hypoglycemia), because its use may be restricted, especially at the late cancer stage that often shows a normal or reduced blood glucose level. There is another possibility to restore the failed insulin secretion in tumor-bearing hosts using the serotonin + tributyre therapy, since the endogenous production of serotonin and gut-derived butyrate in such hosts is seriously reduced. Moreover, the serotonin + tributyre therapy provided leukemic mice with significant survival (41).

The balance points between the two antagonistic hormones (insulin and glucagon) provide a sensitive switch of gluconeogenesis activation during cancer progression. This cancer-bearing state was found to be associated with lowering insulin production (40) and raising secretion of glucagon that is able to promote gluconeogenesis through increased activity of key enzymes responsible for gluconeogenesis (46). As a result, the significantly decreased insulin-to-glucagon ratio is generated. The origin of these cancer-induced hormonal changes in the pancreatic gland remains a mystery. Supposedly, this is a consequence of cancer-mediated damage of pancreatic beta cells with elevated susceptibility to heparanase- or oxidant action, which can be seen in cancers and type 1 diabetes (47). These hormonal abnormalities also occur as a consequence of IR and impaired insulin secretion induced excess glucocorticoids (48). This insulin/glucogon imbalance can be restored by a combination of somastatin and insulin that entails a 23-fold increase of the insulin/glucagon ratio without causing any significant host morbidity from hypoglycemia (43). Notice that plasma glucagon levels are normally suppressed during hyperglycemia but, unexpectedly, are not repressed and might even be slightly increased in some patients with severe IR (49). The excess of glucagon and lack of insulin signaling lead to overproduction of hepatic glucose contributing to the opposite diabetic and cancer cachexia state (1). Therefore, reduction of glucagon secretion can be considered as a key target for reversion of cancer progression that ends with host cachexia. This aim is attained by different ways, one way to decrease the glucagon secretion is to use a neurotransmitter [gamma-hydroxubutyrate (GHB)] produced by pancreatic beta-cells (50). Another way is inhibition of the glucagon receptor with monoclonal antibody, which can restore the blood glucose, IR and GHB levels in diabetes (49), but it is unclear whether this effect works in cancer. Recent data indicate that hyper-glucagonemia can also occur as a consequence of gut-derived glucagon secretion and/or glucagonotropic factor(s) elicited by intraluminal stimulation of the gastrointestinal tract (51). The involvement of this pathway in cancer-stimulated glucagon production is still unclear, although cancer-increased gut permeability occurs frequently (52) and can cause the above mentioned gut hormonal changes.

Not only glucagon but also cancer-induced stress hormones such as glucocorticoids are important because they directly promote hepatic gluconeogenesis that increases the level of blood glucose (53), and by this vicious pathway they can support tumor growth and cancer progression (Figure 2). Glucocorticoids also promote lipolysis producing free fatty acids that induce IR, which is accompanied by a compensatory rise of insulin secretion. This mechanism helps the glucose redistribution from insulin-resistant cells to cancer cells, thus promoting cancer survival and growth (Figure 2). One strategy for targeting glucocorticoid-mediated IR is the use of selective inhibitors of glucocorticoid receptors (54). However, the majority of these inhibitors have side effects that limit their application in cancer patients. It should be noted that the magnitude and duration of stress-induced growth of glucocorticoid hormones can have significant effects on glucose metabolism of the tumor-bearing host. Thus, a short-term stress can enhance the acquisition of anti-tumor immune-protective responses (55). In contrast, a chronic stress can promote cancer development because high amounts of stress hormones lead to formation and functioning of the self-perpetuating vicious cycle (Figures 2, 3) and also suppress anti-tumor immunity.

It is known that chronic inflammation is a key contributor to cancer progression (56), whereas stress-mediated glucocorticoids have an anti-inflammatory property, but both of them can induce IR by cancer development. A simple feedback model of these tumor-host interactions is presented in Figure 2. The cancer-mediated activation of catabolic programs (inflammatory, stress, IR) can create vicious cancer progression initiated by cancer-host imbalance in the glucose delivery and utilization. The proposed model of the vicious cycle involves numerous participants, and each of them may serve as a potential target for the specific anti-cancer treatment. According to this model, a chronic inflammation, an IR-inducing stress, and gluconeogenesis can be considered as attractive therapeutic targets because of their pivotal role in the regulation of glucose metabolism by cancer progression. Metformin is a widely used anti-diabetic drug capable of inhibiting hepatic gluconeogenesis and improving sensitivity; it has demonstrated tumor suppressor properties in many cancer types (1). This is due in part to effectively blocked IGF-receptor activity (57). It will be noted that some malignant tumors and spleen macrophages also release various pro-inflammatory cytokines including IL-6 and TNF-α that impair insulin-mediated glucose uptake and give rise to development of systemic inflammation in tumor-bearing hosts. Intra-peritoneal administration of TNF-α antibody in two mouse models prevented cachexia-associated features, including the loss of white adipocyte tissues and body weight, and significantly reduced the tumor size, thus confirming previous reports on TNF-α involvement in cancer-associated cachexia and IR. Moreover, insulin sensitizers, such as metformin, thiazolidine, and beta2-adrenoreceptor agonists, demonstrate a preliminary ability to increase the muscle mass in catabolic states through activation of components of the insulin signaling pathway (14). The decreased level of blood fatty acids achieved by treatment with pioglitazone alone or in combination with insulin improves IR in tumor-bearing hosts with a small Walker-256 tumor. These results suggest clinical benefits of such a drug combination in preventing IR, adipose tissue wasting and weight loss before tumor progression (36). Dietary administration of the histone deacetylase inhibitor—sodium butyrate microbiota-derived or nutritionally supplemented also can improve IR, causing reduced tumor growth and inhibition of muscle and body weight loss (58).

Taken together, cancer growth and progression is related to an energy and hormonal imbalance. Metabolic re-programming of host cells supports the production of energy-rich glucose via catabolism (through systemic inflammation, stress, and chronic IR), which is then transferred to cancer cells to promote their anabolic growth. This result is achieved by the cancer re-programming of glucose metabolism in distant host organs, thereby creating a cancer-host vicious cycle that may perpetuate its own maintenance as well as cancer growth and progression. The vicious cycle involves numerous participants, and all of them may serve as targets for specific personalized treatment of cancer patients aimed to prevent cancer progression.

## Conclusion

The cancer problem is not merely a cell problem, because it is a problem of cellular interactions not only within cancer-containing tissues, but also within normal cells in other distant tissues. Host-tumor metabolic interactions can be considered as a two-sided process in which cancer cells show the parasitic behavior, because they have no specific function other than growth and dissemination, and compete with the host cells for essential resources such as glucose, lipids and amino acids. This opinion article describes how cancer cells metabolically reprogram the host cells by redirecting the majority of glycolytic fuel from the insulin-resistant host cells to glycolytic cancer cells. In contrast to a transient retention of this metabolic asymmetry without stress and insulin deficit in pregnancy, the chronic retention of such an asymmetry with stress and insulin deficit between cancer and host cells might trigger formation of a vicious cycle (Figures 2, 3); its action is supported by cancer-induced activation of the complex catabolic programs (such as inflammation, stress, and lipolysis) resulting in subversion of the systemic glucose metabolism. This result can be attained by different mechanisms that alter insulin/glucagon/glucocorticoids secretion, host insulin sensitivity and gluconeogenesis, whose synergistic action gives enough glucose for cancer growth through the limited glucose availability for insulin-resistant host cells. Therefore, it would be of great interest to identify the inhibitors of the cancer re-programming of host glucose metabolism. The presented analysis of the vicious cycle action provides new insight into key targets that might be responsible for cancer progression. It is important to further analyze the capacity of these targets in clinical practice for the purpose to prevent the vicious cycle formation and cancer progression.

## Author Contributions

The author confirms being the sole contributor of this work and has approved it for publication.

### Conflict of Interest Statement

The author declares that the research was conducted in the absence of any commercial or financial relationships that could be construed as a potential conflict of interest.
